# Synthesis of graphitic biocarbons from lignin fostered by concentrated solar energy

**DOI:** 10.1038/s41598-025-91204-8

**Published:** 2025-02-21

**Authors:** Salomé Rigollet, Théotime Béguerie, Elsa Weiss-Hortala, Gilles Flamant, Ange Nzihou

**Affiliations:** 1 Mines Albi, CNRS UMR 5302, Centre RAPSODEE, Université de Toulouse, Albi, France; 2 Processes, Materials and Solar Energy Laboratory, PROMES-CNRS, Font-Romeu-Odeillo-Via, France; 3 Andlinger Center for Energy and Environment, Princeton University, Princeton, NJ 08544 USA

**Keywords:** Biocarbon, Carbonization, Concentrated solar, Pyrolysis, Graphitic material, Graphene, Materials for energy and catalysis

## Abstract

The approach aiming at replacing fossil-based carbons by graphitic biocarbon has gained momentum in applications from environmental remediation to battery electrodes and supercapacitors, reducing their environmental impact. To address biocarbon high production temperature and energy consumption, this work uses lignin, a renewable feedstock, and concentrated solar as a sustainable energy source. New insights into lignin’s graphitization mechanism using solar energy are provided. Graphene layers stacking appears as early as 1000 °C in solar carbonization. The structuration and reduction of amorphous carbon was further highlighted at 1400 °C and 1800 °C. At 2000 °C, high graphitic (*L*_*a(XRD)*_ ≈ 9.1 nm, *d*_002_ = 0.3386 nm, 110 stacked layers) and turbostratic (*d*_002_ = 0.3593 nm, 5.5 stacked layers) phases are obtained, showing the structural heterogeneity of solar biocarbon. Contrariwise, conventional biocarbon from electrical heating was homogeneous with limited carbonization at 1800 °C (*L*_*a(XRD)*_ ≈ 3.8 nm, *d*_002_ = 0.3600 nm, 4.4 stacked layers). Textural analysis of solar biocarbons showed aligned graphene layers whereas only random texture was observed on conventional samples. This work established that solar carbonization triggers and enhances graphene layers stacking and growth at lower temperatures whereas conventional carbonization allows the progressive apparition of short graphene layers before stacking and growth.

## Introduction

Graphitic materials (carbon-rich material containing graphite-like structure) have been recently widely used in many applications such as electrodes for batteries^1–3^, supercapacitors^4–6^, for environmental remediation^7–9^ or in energy conversion systems^10–15^. Applications typically depend on the main material properties namely the porosity, the thermomechanical and thermoelectrical, properties among others. Indeed, carbon structure (from amorphous to graphite) directly affects the porosity, electrical or thermal conductivity of the final material, and thus its usage. The quantity and quality of carbons in a graphite structure (stacking of flat graphene layers) influence those properties. For example, graphitic ultra-microporous structures can serve as sieve membranes for hydrogen purification^16^. Biocarbons with a defective graphitic structure (turbostratic) and a high electrical conductivity are useful for electrochemical devices^2^. Since graphitic materials are currently mostly produced from natural graphite^17^, coals, or tars at high-temperature treatment, they benefit in terms of environmental footprint to be produced from an eco-friendlier feedstock such as biomass^2^. Graphitic materials production and their structuration mechanisms (“graphitizable” and “non-graphitizable”) have been widely investigated from fossil-based with high aromatization degree^18,19^. However, reducing the environmental impact of resources and processes is a key issue.

Biocarbon (biochar) are carbon-rich materials produced from high-temperature thermal treatment (carbonization) of bioresource. However, lignocellulosic biomass is classified as “non-graphitizable carbon”^19^ meaning a thermal treatment at temperatures higher than 2000 °C will only give turbostratic structure (defective graphitic structures)^18^. In other words, bio-resource likely produces heterogeneous materials presenting a mixture of different carbon structures including some minerals. Temperature improves the amount of small graphitic domains and decreases the amorphous content. Thus, the process of carbonization of biomass at high temperatures allows the formation of graphitic structures (graphitization mechanism). Biomass graphitization is complex as the mechanism highly depends on the biomass characteristics such as ash and mineral content or its cellulose and lignin content^20^. Studying lignocellulosic biomass macropolymers (cellulose, hemicellulose and lignin) separately allows a better understanding of each contribution to the mechanism. Thus, cellulose valorization into graphitic biocarbon has been largely studied^21–23^. Lignin can also serve for graphitic biocarbon production through pyrolysis or carbonization^24–26^. This resource was selected as a precursor since it already contains carbon aromatic rings (higher aromatization degree than cellulose). However, its composition is highly linked to the original biomass (soft or hard wood) and the extraction process (sulfur or organosolv process for example)^6^. Kraft lignin, which is mainly valorized as an energy source or useful materials^27,28^, was selected in this study to produce highly graphitic biocarbon.

Various techniques are currently developed to produce high-value graphitic materials from bio-resources carbonization, reducing carbon emissions. Highly graphitic biocarbon can be produced from bio-resources below 2000 °C using a catalyst or other pre-treatments^29–32^. Those alternative production routes act on the graphitization mechanism tuning the carbon structure. Still, the most influential operating parameter remains the temperature. This generates a high electrical energy demand for graphitic biocarbon production. A more sustainable alternative for energy consumption can be concentrated solar energy. Concentrated solar carbonization of biomass was done using a parabolic dish concentrator that focuses the incoming solar radiations at a focal point^33^. Such reactors have been widely studied for syngas or bio-fuel production^34–39^. The solid yield is usually low since the carbonization is done at fast (> 60 °C·min^− 1^) or flash (> 1000 °C·min^− 1^) heating rates and presents an interesting carbon organization^40,41^. For example, banana peel solar biocarbons reached an interlayer spacing of 0.381 nm, when exposed to solar radiation over 1000 °C for a few seconds^41^. Solar graphitic biocarbons have also been produced from agave bagasse fibers, agave leaves and tomato peels with promising results for supercapacitor application for samples treated at moderate temperatures (600–900 °C)^40,42^. In another study, solar carbonization of mallee wood produced graphitic biocarbon with a decrease of the interlayer spacing as the temperature increased (1500–2000 °C)^43^. The study of wood pellet carbonization with a solar simulator (xenon lamp) revealed several levels of aromatization and graphitization depending on the distance to the irradiated surface^37,44^. The surface char layer contained graphitic biocarbons while the char layer beneath did not reach the same level of aromatization. Indeed, the high energy focused on the sample’s surface resulted in a high temperature that favored the volatilization of heteroatoms, and then the growth, stacking and flattening of graphene layers^44–46^. Despite those previous works, the solar graphitization mechanism is yet to be described and compared to conventional biomass graphitization.

In this context, this study proposes solar carbonization of kraft lignin as an alternative to conventional carbonization for graphitic biocarbon production. The structure, texture and nanotexture of biocarbons from solar and conventional carbonization were investigated to evaluate the efficiency of solar concentrated energy on lignin graphitization. In addition, the solar graphitization mechanism is explained based on the influence of temperature on the biocarbon graphitic content quality and quantity.

## Results

The initial biocarbon prepared from kraft lignin at 800 °C can be defined as a carbon-rich (C > 85 wt%) material with poor carbon structuration (mostly amorphous carbon). This biocarbon was then processed at high temperature (1000 –1800 °C or 2000 °C) either through conventional carbonization or through solar carbonization. Graphite was only used as a reference. As a reminder, the perfect graphite with *AB* stacking corresponds to a *d*_002_ of 0.3354 nm, while turbostratic structures are commonly characterized with a *d*_002_ of 0.344 nm^19,47^.

### Impact of heating process on carbon organization at bulk scale

**Fig. 1:**
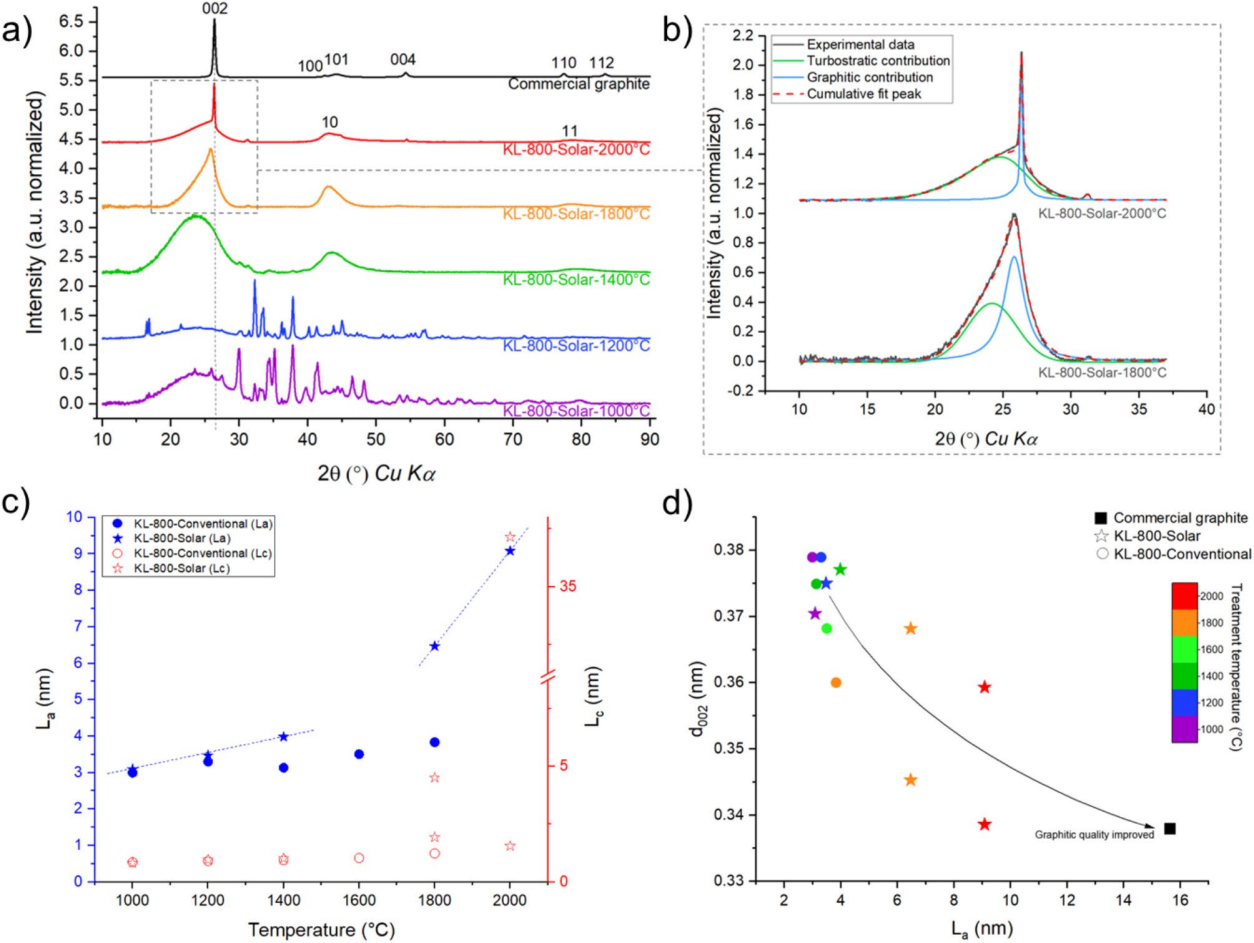
Structure and nanotexture analysis via XRD. (**a**) XRD patterns of samples carbonized between 1000 °C and 2000 °C in solar carbonization and (**b**) curve-fitting of *002* asymmetric peak for samples at 1800 °C and 2000 °C; (**c**) Evolution of dimensions L_a(XRD)_ and L_c_ as a function of temperature and process (dashed lines are used as eye guide); (**d**) d_002_ as a function of L_a(XRD)_.

The crystalline structure of biocarbon was studied using XRD. The general shape of the diffractograms provides information on the presence and quality of graphitic domains. Of particular interest are *002*, *10*, and *11* peaks, from which can be extracted the interlayer spacing (*d*_002_), the crystallite length (*L*_*a(XRD)*_), and the stacking height (*L*_*c*_). For biocarbon from conventional carbonization, the peaks were broad and the *002* peak was symmetrical. Its position at 24.7° was shifted compared to the commercial graphite (26.3 °) due to higher interlayer spacing. This indicated the presence of amorphous carbon, turbostratic, and poor-quality graphitic structures (short layers, high curvature, low number of stacked layers). In comparison, the *002* peak of biocarbon from solar carbonization was asymmetrical, sharper, and shifted (25.8°) towards the graphite position as illustrated in Fig. 1a. This asymmetry accounted for graphitic and turbostratic crystallites when curve-fitted into two peaks^48,49^. Therefore, two values for dimensions *d*_002_ and *L*_*c*_ (both were functions of *002* peak characteristics) were calculated for the solar samples carbonized at 1800 °C and 2000 °C (Fig. 1b).

Figure 1c compares crystallite dimensions from both processes: *L*_*a(XRD)*_, mean crystallite basal diameter and *L*_*c*_, crystallite stacking over temperature. As expected, temperature improved those dimensions, although the process also had an influence. Indeed, Fig. 1c shows higher graphitization of samples from solar heating (stars) compared to conventional electrical heating (circles) at the same temperature. In addition, solar carbonization favored sheet elongation (*L*_*a(XRD)*,_ solid blue star) and stacking (*L*_*c*_, empty red star) above 1200 °C. The *L*_*a(XRD)*_ value of conventionally carbonized samples (blue circles in Fig. 1c) increased by 1.2 times between 1000 °C and 1800 °C whereas, for solar-carbonized samples (blue stars in Fig. 1c), *L*_*a(XRD)*_ was twice higher at 1800 °C than at 1000 °C. This dimension is linked to the average crystallite size of flat and perfect graphene layers^50^. The significant improvement in the mean crystallite diameter *L*_*a(XRD)*_ above 1400 °C illustrated graphene layer growth and graphitic domain flattening induced by solar carbonization. The stacking improvement of solar carbonization was observed at 1800 °C, since the solar biocarbon contains a mixture of large (≈ 5 nm) and small (≈ 2 nm) crystallites while the conventional biocarbon only contains small crystallites (≈ 1.5 nm).

Overall, the solar biocarbon was richer in graphitic structures compared to the conventional one. This improved quality is illustrated in Fig. 1d as crystallite size *L*_*a(XRD)*_ increased while the interlayer spacing *d*_002_ decreased. This highlights a higher proportion of graphitic content in the solar biocarbon. The heterogeneous composition of solar samples at 1800 °C and 2000 °C was also evidenced in Fig. 1d through the two values of *d*_002_. The first structure is a high graphitic structure quality with large crystallites (*L*_*a(XRD)*_ > 6 nm), a high number of stacked layers (*L*_*c*_ > 37 nm at 2000 °C), and low interlayer spacing (*d*_002_ < 0.34 nm at 2000 °C). The second structure is turbostratic and well-disordered with lower *L*_*c*_ values and higher interlayer spacing. In comparison, the mean structure of the sample from conventional carbonization at 1800 °C highlighted turbostratic characteristics without the slightest indication of graphitic structure. The heterogeneity in structure and composition of solar biocarbon could be of high interest for specific applications as it opens the opportunity to synthesize biocarbon materials with desired graphitic content^2,11^, widening the range of applications to be considered. Based on the bulk observation scale, the solar biocarbon contains more graphitic structures at high temperatures. This indicates a difference in carbon organization mechanisms, which could be better investigated at the local scale.

### Impact of the heating mode on the local carbon texture

**Fig. 2:**
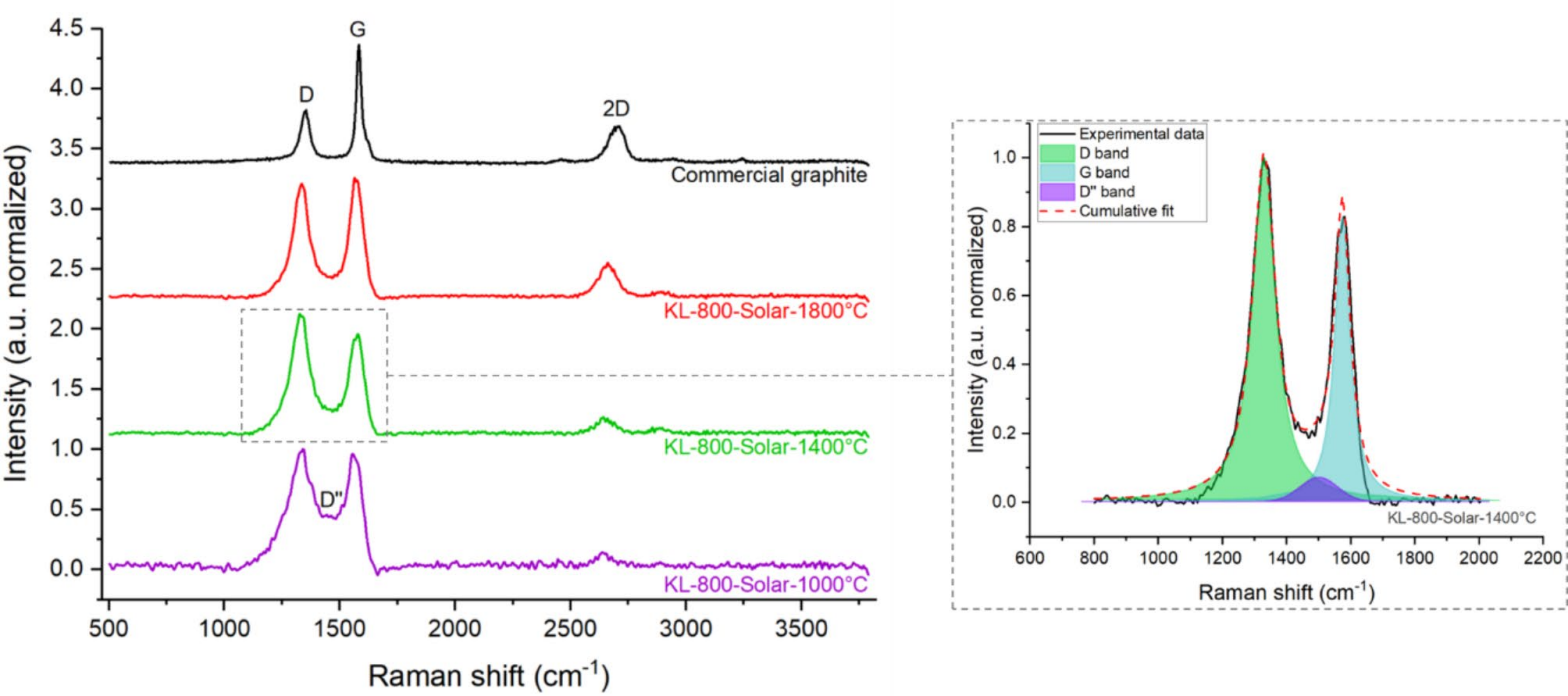
Raman spectra of samples treated in solar carbonized at temperatures between 1000°C and 1800°C with Raman spectra (black) for reference (**left**) and example of curve-fitting into *D*, *G* and *D’’* bands between 800 and 2000 cm^-1^ (**right**).

Data on the texture (distorted or perfect graphene layers) and nanotexture (quality through line defect distance)^50^ of the samples were obtained from Raman spectroscopy. The spectrum gave information on the crystallinity, presence of defects, and line defect distance in biocarbon^22^. For graphite, three bands are of interest: the *G* (1580 cm^− 1^) and *D* (1350 cm^− 1^) bands, respectively attributed to graphene layers and defects, and the *2D* band (2700 cm^− 1^) indicating well-stacked graphene layers. For graphitic biocarbon, a fourth band, *D’’*, appears due to the presence of amorphous carbon. This forms a valley between the *G* and *D* bands (around 1495 cm^− 1^). To investigate the graphitization of lignin in both processes, these four bands, and their corresponding ratios, were considered. Raman spectra of solar carbonized samples are presented in Fig. 2 as well as an example of curve-fitting of *D*, *G* and *D’’* bands.

#### Defects in the graphitic arrangement

According to the literature^51,52^, most carbon materials exhibit a *D* band, however its physical interpretation depends on the Raman spectrum shape. For our solar samples, the absence of shoulders and the low valley intensity let us interpret the *D* band as defects such as graphene layers edges (line defect), vacancy or heteroatom presence (point defect), and curvature (turbostratic structure)^53,54^. The samples were compared using the intensity ratios (such as *I*_*D*_*/I*_*G*_ ratio). Along the carbonization process, the *I*_*D*_*/I*_*G*_ ratio first increased with the development of isolated graphene layers or very small (nano) graphitic domains. As the carbonization progressed, the amount and the quality of graphitic domains increased leading to a decrease of this ratio^55,56^.

For conventional heating, *I*_*D*_*/I*_*G*_ ratio increased by 39% due to an increase in the intensity of the *D* band between 1000 °C and 1800 °C. The *D* band is correlated to various types of defects from curvature to line or point defects, with various intrinsic intensities^54^. The increase in *D* band intensity was most probably caused by the growth of new defective short graphene layers (multiplied edges defects). With solar heating, an increase of 5% in *I*_*D*_*/I*_*G*_ ratio was observed between 1000 °C and 1800 °C. This reflects a slight imbalance in the growth of defective graphene layers over defect reduction.

#### Amorphous content and graphitic character

In biocarbon with a certain carbon organization such as our samples, the valley (*D’’* band) mainly represents the amorphous carbon. The *A*_*D’’*_*/A*_*Total*_ ratio of integrated areas decreased with temperature, showing the consumption and/or structuration of amorphous carbon. This was seen in both processes at 1800 °C, with *A*_*D’’*_*/A*_*Total*_ ratio of 0.02 and 0.03 for conventional and solar respectively. It was further confirmed by the presence and intensity of the *2D* band. In solar carbonization, the *2D* band appeared from 1000 °C, showing the presence of well-stacked layers and the *A*_*2D*_*/A*_*Total*_ ratio increased by 74% between 1000 °C (*A*_*2D*_*/A*_Total_ = 0.06) and 1800 °C (*A*_*2D*_*/A*_Total_ = 0.10). For conventional carbonization, the *2D* band appeared at 1400 °C with *A*_*2D*_*/A*_Total_ ratio of 0.05 which reached 0.10 at 1800 °C. This result was consistent with the analysis of broad and shifted peaks in XRD: biocarbon samples produced from conventional carbonization present a large portion of amorphous and disorganized structures whereas solar-biocarbon samples contain a larger part of graphitic structures.

#### Mechanisms

**Fig. 3:**
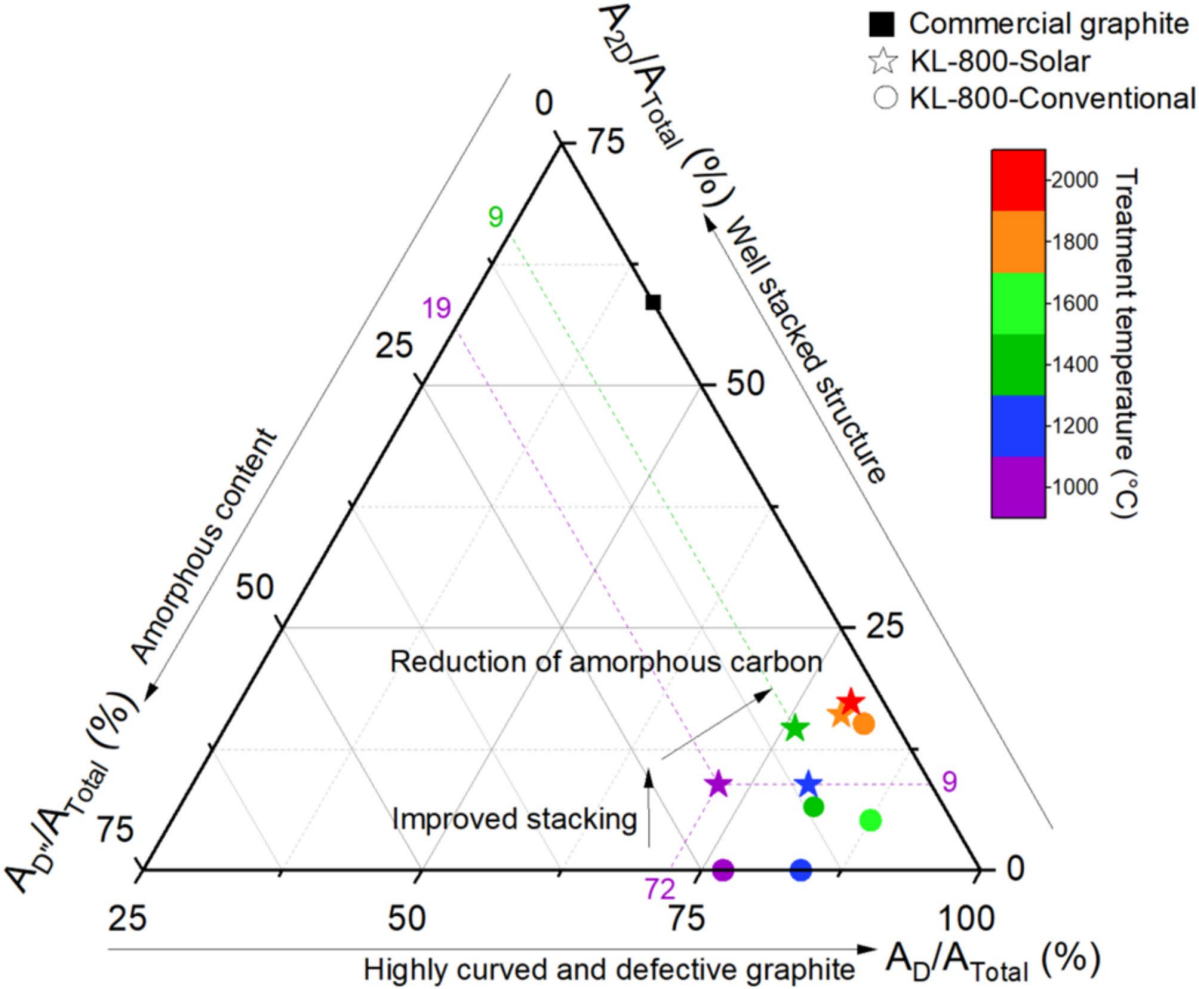
Overview of main structural changes observed in Raman spectra of solar-carbonized (stars) and conventionally carbonized (circles) kraft lignin samples.

A ternary representation was used from Raman data to better account for the changes in biocarbon structures (Fig. 3). The area ratios of the bands *D*, *D’’* and *2D* integrated over the total area were calculated and weighted to highlight the major effect. Ratios were then normalized to be plotted in a ternary diagram using Eq. (1).

1 $$\frac{{A}_{D}}{{A}_{Total}}+\frac{{A}_{2D}}{{A}_{Total}}+\frac{{A}_{D{\prime}{\prime}}}{{A}_{Total}}=100{\%}$$

The diagram features the results obtained from commercial graphite (black square in Fig. 3), as a reference. Raman spectrum of graphite samples is characterized by an intense *G* band, a weak *D* band, without a valley between them, and a *2D* band. Finally, the commercial graphite, which does not contain amorphous carbon, was plotted on the triangle side of the *A*_*2D*_*/A*_*Total*_ ratio, where *A*_*D’’*_*/A*_*Total*_ ratio was equal to zero, the *A*_*2D*_*/A*_*Total*_ ratio accounts for 59% and the *A*_*D’’*_*/A*_*Total*_ ratio for 41%. Biocarbons that did not show a *2D* band in their Raman spectra were plotted along the *A*_*D*_*/A*_*Total*_ axis where *A*_*2D*_*/A*_*Total*_ ratio is equal to zero. As a simplified overview, the graphitic content increases when moving toward the top of the triangle, and the amorphous content decreases when moving rightwards.

For kraft lignin biocarbon treated in a conventional carbonization furnace (circles in Fig. 3) at 1000 °C and 1200°C, no *2D* band appeared. These two samples were plotted on the side of *A*_*D*_*/A*_*Total*_ ratio. The right shift of the 1200°C sample indicates a reduction in the amorphous content with temperature, as expected. The Raman signature, and its description on this ternary figure agree with XRD data. Indeed, it was concluded that the carbon was mostly amorphous or highly turbostratic, with a high interlayer spacing of 0.3789 nm. At 1400°C, the point moves away from the base of the triangle, indicating the stacking of graphene layer (apparition on Raman spectra of a *2D* band), and the right shift indicates a reduction of the amorphous content (lower valley). From 1400°C to 1600°C, the A_*2D*_*/A*_*Total*_ ratio was almost similar, while the *A*_*D’’*_*/A*_*Total*_ ratio decreased and the *A*_*D*_*/A*_*Total*_ ratio increased. Therefore, stacking was not the main mechanism involved in the structure transformation from 1400 °C to 1600 °C. This agrees with the XRD data, showing a moderate gain of 11% in *L*_*c*_ value (see Fig. 1c). The amorphous content decreased from 1400 °C to 1600 °C, meaning that new graphite crystallites were produced. This results in increasing the proportion of various defects: new edges, point defects, or curved layers. Finally at 1800 °C, the diminution of interlayer spacing combined with the *L*_*c*_ increase (see Fig. 1) induced better stacking, which was confirmed by a relative increase in the *2D* band intensity. The highest temperature allowed the simultaneous reduction of amorphous content and stacking increase.

For kraft lignin biocarbon produced by solar carbonization (stars in Fig. 3), two steps can be differentiated. It is worth noting that all the samples exhibited well-stacked structures, supported by the presence of the *2D* band. From 1000°C to 1200°C, amorphous content decreases resulting in a right shift. After solar treatment at 1200°C, the stacking and amorphous content are similar to the sample from conventional carbonization at 1400°C. This result agrees with XRD data, *L*_*a(XRD)*_ ≈ 3 nm and *d*_002_ ≈ 0.37 nm for those two samples, exhibiting the efficiency of solar carbonization at low temperature. From 1200°C to 1400°C, the most affected contribution was related to amorphous carbons *(A*_*D’’*_*/A*_*Total*_ ratio), while stacking contribution increased slightly and defects in graphene sheets contribution decreased. Indeed, the contribution of the *A*_*D’’*_*/A*_*Total*_ ratio decreased from 19 to 9%. The increase of stacking proportion (from 9 to 14%) is consistent with XRD values that only integrated a moderate *L*_*c*_ increase (+ 11%).

Between 1400 °C and 1800 °C the solar graphitization mechanism reduced the contribution of amorphous carbon (from 9 to 4%), while the proportion of stacking was almost similar. This is in agreement with XRD data, which showed a heterogeneous material at 1800 °C with two different phases (average *L*_*c*_ of 2 and 4.5 nm). Since the proportion of carbon organized in aromatic rings increased, the ratio of defects increased (*A*_*D*_*/A*_*Total*_ ratio) from 76 to 79%. From 1800 °C to 2000 °C the ongoing transformations run smoothly (see Fig. 3).

Overall, solar heating triggered the stacking and growth of graphene layers at lower temperatures because of the focused and high energy density whereas conventional carbonization allowed the progressive apparition of short graphene layers before stacking and growth.

#### Nanotextural investigation

*L*_*a(Raman)*_ values obtained from the Raman spectra refer to the distance between two crystallites separated by a line defect^50,54^. This dimension was calculated using the Tuinstra and Koenig Equation ^57^ (3), from the *I*_*D*_*/I*_*G*_ ratio. In conventional carbonization, the intensities of *D* and *G* bands were similar up to 1400 °C, resulting in *L*_*a(Raman)*_ values of 4.8–4.3 nm (Table 1). These values were higher than those calculated from XRD (*L*_*a(XRD)*_ ≈ 3 nm (Fig. 1c). This was expected since *L*_*a(XRD)*_ measures the mean diameter of flat crystallites while *L*_*a(Raman)*_ corresponds to the distance between two in-plane defects^50^. Raman spectra of samples treated at 1600 °C and 1800 °C in a conventional furnace showed an intense *D* band. The *D* band intensity is the sum of the intrinsic intensity of each defect weighted by its rate. There are some great differences in the intrinsic intensity of those defects that might significantly affect the total intensity^56,58,59^. An intense *D* band indicates an increase in the proportion of defects in the biocarbon. The increase of the *I*_*D*_*/I*_*G*_ ratio resulted in a decrease in *L*_*a(Raman)*_ values, and the distance between two defects (edges or in-plane) was reduced by 29% at 1800 °C compared to the 1000 °C sample. At this local scale, the selected areas were composed of short, probably curved graphene layers. Thus, in conventional carbonization, the mean length of flat crystallites increased from 2.99 nm at 1000 °C to 3.83 nm at 1800 °C (*L*_*a(XRD)*_), while the local distance between two defects decreased from 4.88 nm at 1000 °C to 3.48 nm at 1800 °C (*L*_*a(Raman)*_).

For samples produced by solar carbonization, the heating process favored the flattening and stacking of graphene layers with a significant increase of flat crystallite size *L*_*a(XRD)*_ from 3.09 nm at 1000 °C to 6.47 nm at 1800 °C (the mean diameter was doubled). The dimension extracted from Raman spectra is directly linked to the relative intensity of the defect band. For solar samples, this relative intensity varied with temperature and resulted in a slight modulation of *L*_*a(Raman)*_: + 7% between 1000 °C and 1400 °C, and − 11% between 1400 °C and 1800 °C (see Table 1). As seen in Fig. 3, the proportion of defects increased at 1800 °C, which explains a decrease in the distance between two defects.

**Table 1:** L_a(Raman)_ values of solar and conventionally carbonized kraft lignin samples calculated using Tuinstra and Koening equation.

	Temperature (°C)	Mean L_a(Raman)_ (nm)
LK-800-conventional	1000	4.88 ± 1.03
	1400	4.30 ± 0.15
	1800	3.48 ± 0.68
LK-800-solar	1000	5.43 ± 0.28
	1400	5.80 ± 1.81
	1800	5.18 ± 0.43

**Fig. 4:**
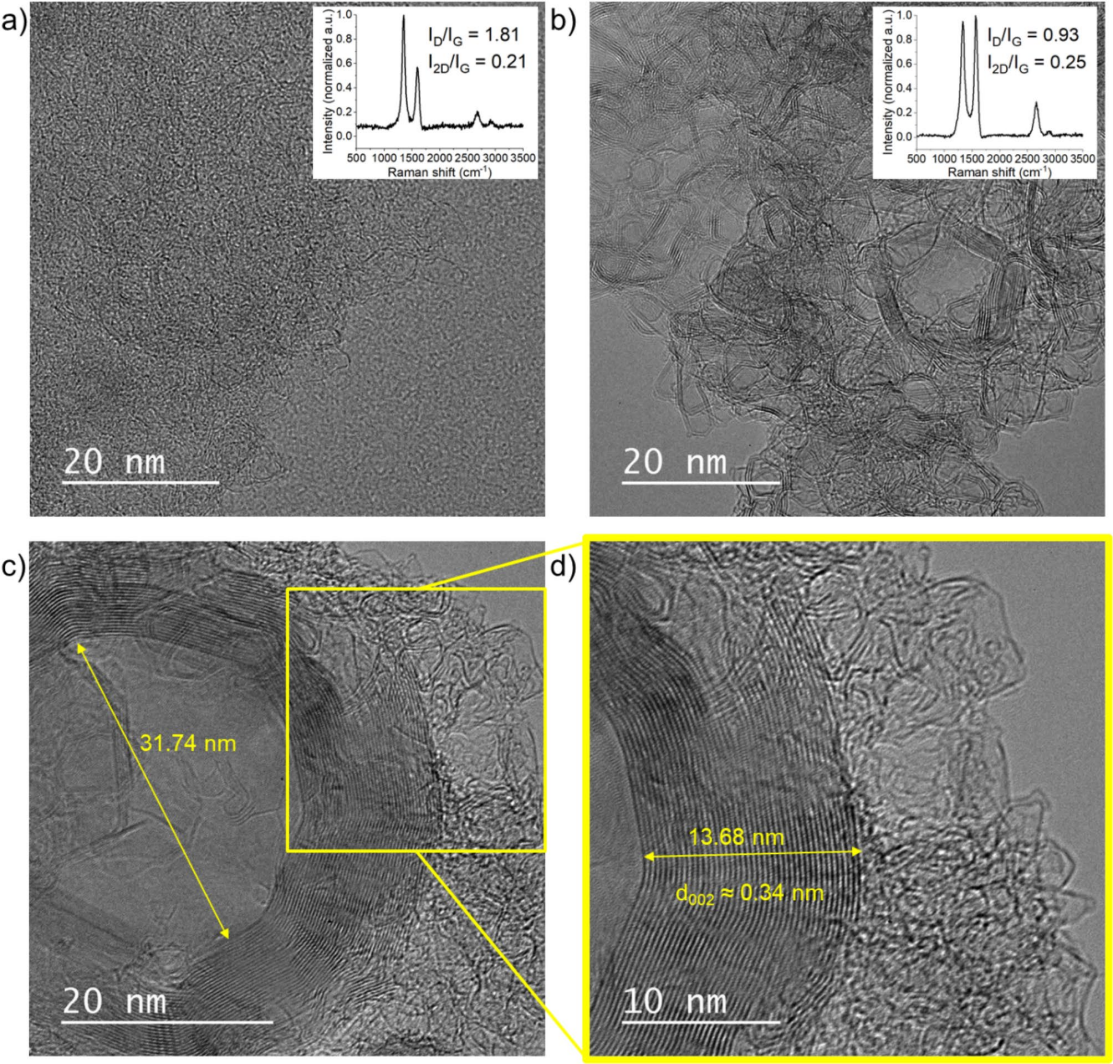
Texture analysis. (**a**) TEM image of sample KL-800-Conventionnal-1800 °C (scale is 20 nm and magnification is 300,000x) and insert its Raman spectrum, (**b**) HRTEM image of sample KL-800-Solar-1800 °C (scale is 20 nm and magnification is 300,000x) with insert its Raman spectrum and (**c**) HRTEM image of sample KL-800-Solar-1800 °C (scale is 20 nm and magnification is 400,000x) and (**d**) zoom-in on graphene layer stacking (scale is 10 nm and magnification is 600,000x).

A visual description of the defects is requested, such as using TEM images, to better describe the evolution of *L*_*a(Raman)*_. The image of biocarbon obtained after conventional carbonization (Fig. 4a) showed highly curved layers, random organization of short-range graphene layers, and poor stacking. These characteristics explain the shape of the corresponding Raman spectrum (Fig. 4a insert) with a high *D* band (*I*_*D*_*/I*_*G*_ = 1.76). For the solar-heated biocarbon (Fig. 4b), the sample contained a large variety of textures. Both highly curved and flat layers were observed. The presence of well-stacked graphene layers was consistent with an intense *2D* band in Raman spectrum (Fig. 4b insert) reaching *I*_*2D*_*/I*_*G*_ ratio of 0.23. In the solar sample, graphene layers were highlighted, and their curvature and edges contributed to the *D* band intensity. Although TEM images are a 2D-projection of the biocarbon network, the main organization seems to be composed of concentric structures (Fig. 4c). The inner diameter of those structures varies between 12 and 35 nm. Smaller structures (cavities) with diameters of 2 to 6 nm composed of two graphene layers also appear (Fig. 4b). A deeper analysis (Fig. 4d) of this 1800 °C solar sample gave a local estimation of the interlayer distance of about 0.34 nm. Based on more HRTEM images, *d*_002_ value ranged from 0.35 nm to 0.34 nm in stacked domains of 5 to 40 layers. Those estimations are consistent with XRD values calculated with the two components of the *002*  peak (1800 °C, solar sample): 0.345 nm and 0.375 nm.

The HRTEM images confirmed the overall beneficial effect of solar carbonization on lignin graphitization at 1800 °C, previously observed through XRD and Raman spectroscopy. It showed that solar carbonization leads to the formation of well-organized and curved graphitic structures in lignin biocarbon.

## Discussion

The analysis carried out from bulk to nano scales revealed the graphitization efficiency of solar concentrated carbonization on kraft lignin biocarbon.

**Fig. 5:**
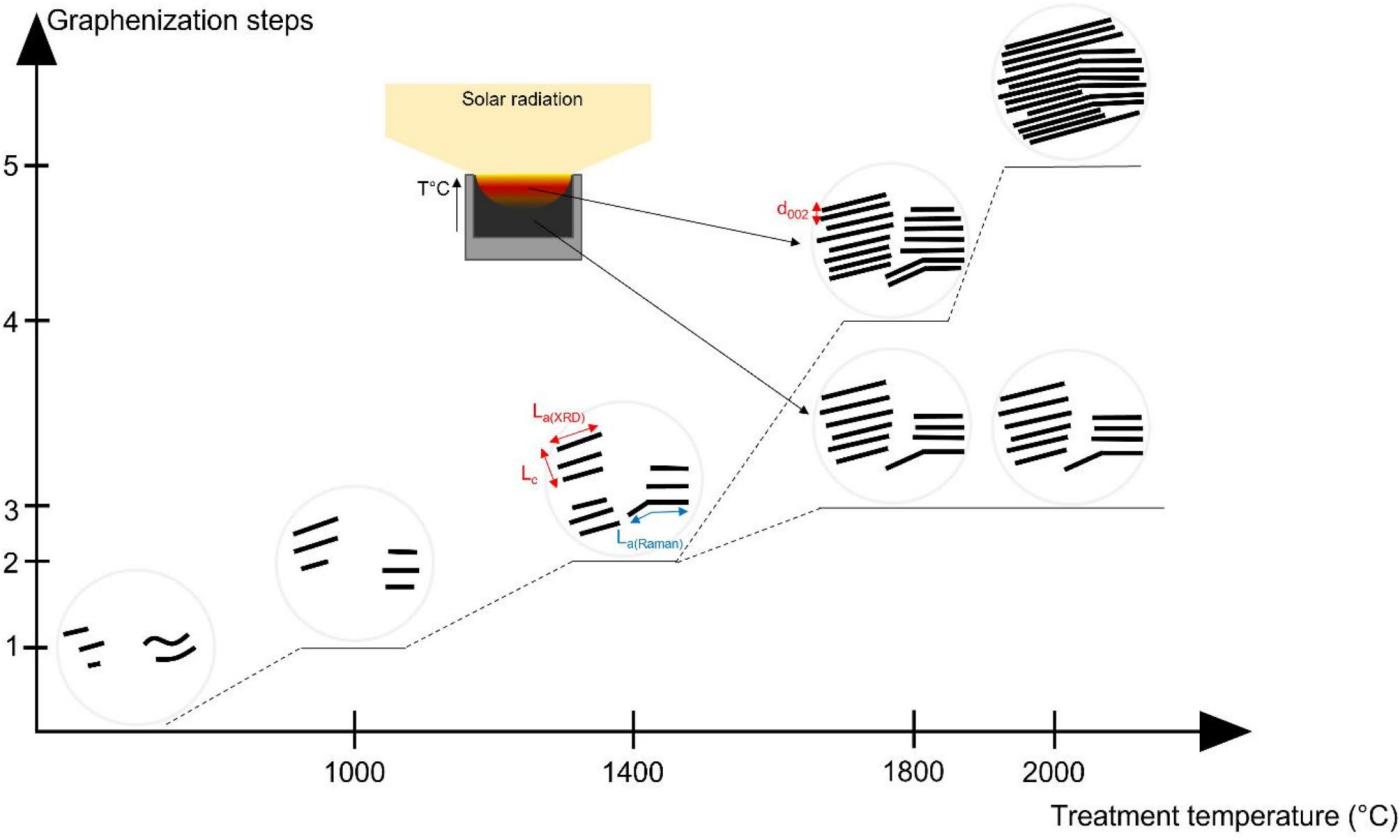
Solar graphitization mechanism.

Solar carbonization is an efficient process for biocarbon graphitization. The resulting heterogeneous material was composed of graphitic and turbostratic (distorted graphite) structures. Figure 5 illustrates the different steps of the biocarbon graphitization over the temperature in the solar reactor, according to the scheme proposed by Oberlin for coke graphitization^18^. As a reminder, Oberlin^18^ defined 4 stages of the graphitization mechanism: small structural units (stage 1, *L*_*a(XRD)*_ ≈ 1 nm, 3 stacked graphene layers) stack into distorted columns at about 500 °C (stage 2, *L*_*c*_ increase) and continue to coalesce into distorted layers from 1500 °C (stage 3, *L*_*a*_ increase) to finally flatten into graphitic carbons around 2000 °C (stage 4, significant *L*_*a(XRD)*_ increase). The feedstock in this study (bottom left in Fig. 5) is a carbon-rich material with poorly ordered carbon and high interlayer spacing value (high *d*_002_). According to the XRD characterizations, the solar biocarbon was composed of a few short graphene layers with poor stacking when treated at 1000 °C (reaching step 1 of graphitization in Fig. 5). The crystallite mean diameter (*L*_*a(XRD)*_ ≈ 3.1 nm) was slightly larger than the stage 1 structural units as defined by Oberlin^18^, but the high *d*_002_ value (0.3704 nm) accounted for a large proportion of amorphous carbon (with no crystalline organization). At 1400 °C, the crystallite dimensions did not significantly change, leading to 3.7 stacked graphene layers and a mean crystallite diameter of 4.0 nm (*L*_*a(XRD)*_). As mentioned previously, *L*_*a(Raman)*_ was slightly larger (5.8 nm) since it also counts curved graphene layers length. However, at this temperature the amorphous content decreased leading to almost the same structure but in higher proportion (step 2 in Fig. 5). Indeed, at 1400 °C, characteristic dimensions of solar biocarbon (*d*_002_ = 0.3771 nm) were slightly higher than conventional biocarbon (*d*_002_ = 0.3749 nm), while the amorphous content significantly decreased. Thus, graphene layer growth was the main phenomenon at this temperature range (similar *L*_*c*_ values). Moving to 1800 °C, highly focused solar energy led to an increase of the graphitic rate in the material (step 4). This was highlighted by a significant decrease in the interlayer spacing accompanied by a high number of stacked long graphene layers (step 4, *L*_*a(XRD)*_ ≈ 6.5 nm, *d*_002_ = 0.3453 nm, *L*_*c*_ ≈ 4.51 nm, corresponding to about 14 stacked layers). However, a second phase, composed of distorted graphite (turbostratic structure), characterized the solar biocarbon (step 3, about 6 stacked layers). This difference in the heterogeneous carbon material could be explained by the temperature gradient in the sample during irradiation. This phenomenon was observed by Zhong et al.^37^ with physical and chemical structure change between layers of a wood pellet under solar flux. At 2000 °C, the solar flux focused on the surface led to an increase in the crystallite dimensions (step 5, *L*_*a(XRD)*_ ≈ 9.1 nm, *d*_002_ = 0.3386 nm, *L*_*c*_ ≈ 37 nm, resulting in about 110 stacked layers). Both phenomena, stacking, and graphene layer growth, were involved in the carbon structuration at the surface. In the core of the biocarbon, the structure was close to step 3 (5.5 stacked layers). This means that graphene layer growth was the main phenomenon. In comparison, conventional biocarbon reached an organization close to step 2, with a lower interlayer distance (at 1800 °C, *L*_*a(XRD)*_ ≈ 3.8 nm, *d*_002_ = 0.3600 nm, *L*_*c*_ ≈ 1.2329 nm, corresponding to about 4.4 stacked layers).

The heterogeneous composition of the solar biocarbon at high temperature can be explained by the heating process. In the solar carbonization process, the sample was rapidly heated by a high-flux radiation at its surface while in conventional carbonization convection and low-flux density infrared radiation were the dominant heating processes. Since concentrated solar radiation was highly localized, the main heating process was conduction in the sample resulting in the establishment of a temperature gradient in the sample. The advantage of such high energy was the direct influence on the physical transformation of carbon structures in the material that easily and rapidly arranged in stacks of graphene layers at much lower temperatures (1800 °C, step 4) than conventional graphitization (above 2000 °C). However, the heat dissipation created zones in the sample with different treatment temperatures leading to heterogeneous structures^44^. This material could develop interesting properties in terms of electrical and thermal conductivity or magnetic properties that might not be reached by a conventionally carbonized biocarbon. Indeed, electrical conductivity is likely improved with the basal plane growth (*L*_*a(XRD)*_), while thermal conductivity also benefits from a multi-layer domain. In terms of form factor, biocarbon in step 4 would be preferred with a *L*_*a(XRD)*_*/L*_*c*_ ≈ 1.4 compared to step 5 biocarbon (*L*_*a(XRD)*_*/L*_*c*_ ≈ 0.25), as long as these crystallites are in high proportion and the distance between them is small to facilitate electron hopping^60–62^.

Heterogeneous biocarbon was easily produced with concentrated solar energy. However, because of the temperature gradient and the heating rate, the biocarbon structure could not be finely controlled. In addition, the graphene layers stacked in concentric or cavities structures which might indicate a catalytic effect of inherent metal particles. The combination of such technique with a catalyst should be efficient, as initiated in several works^63,64^, and highlight the versatility of such materials in terms of applications^14,42,65^.

## Conclusion

Graphitic biocarbon was produced from kraft lignin using conventional carbonization in an electrical furnace and solar carbonization in a concentrated solar reactor. The high energy density focused on the sample surface in solar carbonization was revealed to be efficient in promoting graphitic content in high quantity (higher proportion of graphitic carbon over amorphous) and high quality (longer graphene layers, lower interlayer spacing, high number of stacked graphene layers) in biocarbon as compared to biocarbon from conventional carbonization.

Temperature affected the structure of biocarbon with an increase of graphitic and distorted graphite (turbostratic) structures and a reduction of amorphous content. At the bulk observation scale, solar carbonization (high heating rate) significantly improved the texture and nanotexture of biocarbon. In the temperature range of 1000 –2000 °C, biocarbon evolved as follows.

- From 1000 °C to 1400 °C: the biocarbon structure and nanotexture did not evolve much. The size of the small crystallites was almost similar (*L*_*a(XRD)*_ ≈ 3.1–4.0 nm) but the amorphous content decreased leading to the same structure in higher proportion. At the same time, the stacking was slightly improved.
- From 1400 °C to 1800 °C, two phases were produced: graphitic structures (*L*_*a(XRD)*_ ≈ 6.5 nm, *d*_002_ = 0.3453 nm, *L*_*c*_ ≈ 4.51 nm, corresponding to about 14 stacked layers) and turbostratic structures. This heterogeneity came from the temperature gradient in the sample during irradiation. For the graphitic phase, stacking and layer growth phenomena were involved.
- At 2000 °C, highly graphitic biocarbons were obtained (*L*_*a(XRD)*_ ≈ 9.1 nm, *d*_002_ = 0.3386 nm, *L*_*c*_ ≈ 37 nm, resulting in about 110 stacked layers). A second phase was composed of less organized structures. At the focal point, stacking and graphene layer growth was still the main phenomena involved.

For biocarbon produced from conventional heating, the stacking was improved with the temperature (1000 –1800 °C), especially in terms of interlayer spacing, while the growth of graphene layers was quite limited (at 1800 °C, *L*_*a(XRD)*_ ≈ 3.8 nm, *d*_002_ = 0.36 nm, *L*_*c*_ ≈ 1.23 nm, corresponding to about 4.4 stacked layers).

Local observations with Raman spectroscopy (presence of *2D* band, no shoulders, low valley) and HRTEM images confirmed the presence of wide graphitic domains in solar biocarbons. Indeed, aligned graphene layers were observed by HRTEM for solar samples whereas only random texture and crumpled morphology can be seen on conventionally treated samples. Finally, the local concentration of solar rays provided sufficient and well-focused energy that promoted the stacking and growth of graphene layers. This heating method generated a local temperature gradient that resulted in heterogeneous materials. The heterogeneity of solar biocarbon containing both graphitic and turbostratic structures represents a great opportunity to synthesize a wide variety of materials with diverse properties for given applications. Both conventional and solar biocarbons obtained at high temperature could be of interest for anode material in electrochemical and electrical applications (batteries, supercapacitors, sensors among other applications) as a replacement for fossil-fuel-based carbon materials currently used^3^. Ongoing studies will focus on the characterization of pore structure and volume, electrochemical properties as well as X-ray photoelectron spectroscopy (XPS) for a better identification of surface functionalities and defects^23^.

## Materials and methods

### Biocarbon production

Kraft lignin (Sigma Aldrich CAS: 8068-05-1) was first pyrolyzed in a vertical tubular oven (Carbolite tubular furnace) at 800 °C for 1 h with a heating ramp of 2 °C·min^− 1^ and under N_2_ gas flow of 1 L.min^− 1^. The resulting biocarbon was collected and heated up to 1000–1800 °C or 2000 °C for 1 h in either a conventional electric furnace or a solar reactor.

The electric tubular furnace (Nabertherm RHTH 80/300/18) had a heating ramp of 2 °C min^− 1^ and a gas flow rate of 7 L.min^− 1^ of N_2_. The maximum temperature studied is 1800 °C, to limit the energy consumption^29^.

**Fig. 6:**
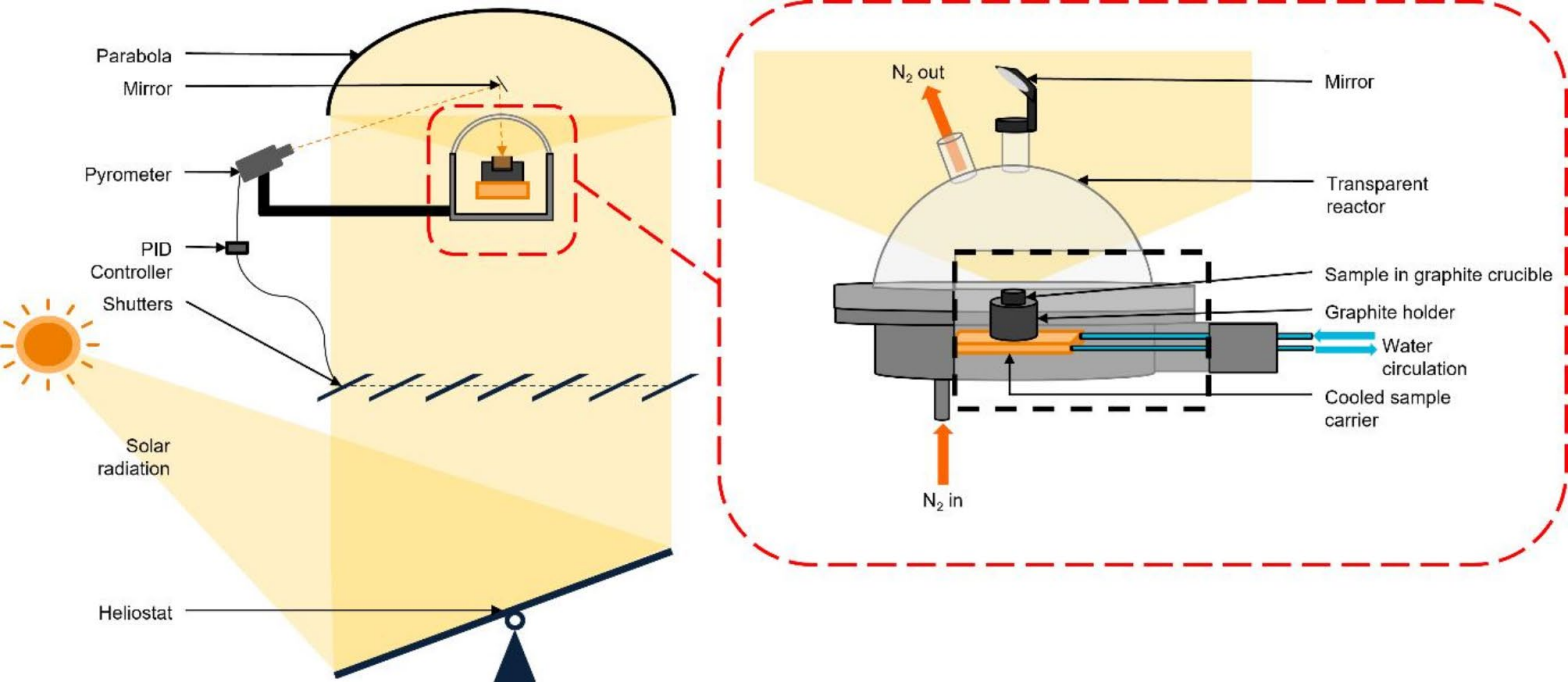
Schematic of the solar pyrolysis experimental setup.

The solar furnace consisted of a tracking heliostat reflecting the incident solar flux vertically towards a parabola. The 2 m diameter parabola concentrated the solar flux onto a 12 mm diameter spot. Since reaching high temperature does not require additional electrical input, the maximum temperature studied is 2000 °C, the end of secondary carbonization. The temperature of the sample at the focus point was controlled using a solar-blind optical pyrometer (KLEIBER monochromatic at 5.2 μm) and a system of shutters monitored by a proportional-integral-derivative (PID) controller adjusting the solar flux coming from the heliostat to the parabola. The sample emissivity was measured before the experiment using a SOC-100 HDR reflectometer (Surface Optics Corporation Hemispheral Directional Reflectance) coupled with an IS50 FTIR spectrophotometer operating in the range from 1.25 μm to 25 μm. The reactor consisted of a metallic base on which a transparent dome was placed. This dome was equipped with optical ports and swept by N_2_ (3 L.min^− 1^). The 12 mm diameter graphite crucible was put on a water-cooled sample holder. An insulation layer was inserted between the crucible and the sample holder to limit the temperature gradient. The reactor was positioned to place the sample at the focal point (see Fig. 6). The heating ramp was 1200 °C·min^− 1^ and the temperature was held for 1 h. For each temperature, the experiment was repeated at least three times, and the samples were mixed to avoid heterogeneity and average potential experimental errors (temperature, emissivity and sampling).

The samples were named first the initial feedstock (kraft lignin abbreviated KL), the intermediate pyrolysis treatment (800 referring to the temperature), the type of high-temperature treatment (solar or conventional) and the final treatment temperature (between 1000 and 2000 °C). For instance, sample *KL-800-solar-1800* is kraft lignin, pre-treated with pyrolysis at 800 °C and then carbonized in the solar reactor at 1800 °C.

A commercial graphite (ChemPur CAS: 7782-42-5) was used as a reference for its graphitic structure and texture.

### Characterization of biocarbon at various observation scales

The structure of biocarbon was studied using X-ray diffractometry (XRD). The instrument was an Empyrean series 3 PANanalytical X-ray diffractometer, using CuKα radiation source (*λ* = 0.1542 nm), operating at 45 kV and 40 mA. The recorded spectral range was between 10 ° and 90 ° in 2θ with a measurement step of 0.03 ° and a scan speed of 0.02 °. s^− 1^. The resulting diffractograms were fitted using relevant functions: pseudo-Voigt for *002* peak fitting and Breit-Wigner-Fano for *10* and *11* peaks^22,49^. When the *002* peak presented a shoulder, two pseudo-Voigt curves were used as illustrated in Fig. 1a^49,53^. The dimensions *L*_*a(XRD)*_ (in-plane length) and *L*_*C*_ (stacking height) of the crystallites were calculated using the Scherrer Eq. (2).

2 $${L}_{a,c}=\frac{K\times\lambda}{\sqrt{{\beta}^{2}-{s}^{2}}\times\text{cos}\theta}$$

${L}_{a,c}$ and $\lambda$ (radiation wavelength) is expressed in nm. The full width at half maximum of silica, $s$, was used as a standard specimen to adjust for instrument broadening. For *L*_*a(XRD)*_, the Bragg angle $\theta$ and the full width at half maximum $\beta$ were taken from the *10* peak and the correcting factor $K$ is equal to 1.84. For *L*_*c*_, $\theta$ and $\beta$ were extracted from the *002* peak and $K$ is equal to 0.89. The *K* values are commonly used in the literature to describe these bands^50,66^.

The interlayer spacing *d*_002_ was determined using Bragg Eq. (3). $\lambda$ is the radiation wavelength and ${\theta}_{002}$ is the Bragg angle of the *002* peak.

3 $${d}_{002}=\frac{\lambda}{2\times\text{sin}{\theta}_{002}}$$

The samples’ nanotexture was studied using a Raman confocal microscope WITec Alpha 300R with a 532 nm/ 2.33 eV laser. Tablets of about 2 wt% of biocarbon mixed in KBr were used to avoid fluorescence and to dissipate heat. The analyzed surface was a square of 36 μm^2^ area with 30 lines per image and 30 points per line. Two sites were analyzed per sample and for each, a cluster option highlighted the spatial distribution of various Raman signatures when relevant.

The Raman spectrum of graphite presents two main bands: *D* band (for defects) and *G* band (for graphitic structures). Along the graphitization mechanism of biomass, the shape and size of those two bands evolve due to the presence of amorphous carbon and various defects in the graphene layers^53,54^. To account for those effects, Raman spectra are usually fitted with 4 or 5 bands^53,67,68^. The 5 bands account for distorted graphitic lattice (shoulders on *D* and *G* band), amorphous carbon (valley between *D* and *G* band), defect (*D* band), and graphitic structures (*G* band)^67^. The Raman spectra of solar samples did not highlight shoulders on *D* and *G* bands. Thus, the spectra were fitted in 3 bands: *D*, *D’’* and *G* corresponding respectively to defects in graphitic structures, amorphous carbon, and graphitic structures. An example of the 3 bands fit is presented in Fig. 2. The *2D* band (2700 cm^[-1^), which arises from graphitic structures and perfect stacking, was also fitted as detailed in Table 2. The integrated area of each band (*A*_*D*_, *A*_*D’’*_, *A*_*G*_ and *A*_*2D*_) gave structural information on the composition of the material.

**Table 2:** Fitting parameter for Raman spectra analysis of biocarbon samples.

Band	Approximate position (cm^− 1^)	Structural information	Curve fitting
D	1350	Defects in graphitic structure	Lorentz
D’’	1495	Amorphous carbon	Gaussian
G	1580	Graphitic structures	Lorentz
2D	2700	Well-stacked graphitic structures	Gaussian

The length of the graphene layer, *L*_*a(Raman)*_, can be extracted from the ratio of *G* and *D* bands intensities. However, this dimension is influenced by carbon hybridization, resulting in a non-linear relation with this ratio^52^. For highly amorphous carbon, containing mainly carbons with sp^3^ hybridization, *L*_*a(Raman)*_ is well described using the Ferrari and Robertson relation up to 2 nm^52^. Beyond that value, turbostratic to graphitic materials, such as our samples, are better described with the Tuinstra and Koenig Equations^57,69,70^. Thus, *L*_*a(Raman)*_ was calculated using Eq. (4). This dimension corresponds to the distance between two line defects in graphene layers^51,54^.

4 $${L}_{a\left(Raman\right)}=\frac{4.4}{\frac{{I}_{D}}{{I}_{G}}}{\left(\frac{2.41}{{E}_{L}}\right)}^{\alpha}$$

${E}_{L}$ is the laser energy and $\alpha$ was taken as 4 (graphite value)^69^. $\frac{{I}_{D}}{{I}_{G}}$ is the ratio of intensities of the *D* band over the *G* band.

Texture and morphology information were obtained using transmission electron microscopy (TEM). The microscope (JEOL JEM-ARM200F Cold FEG with probe Cs corrected) operated at 200 kV in scanning TEM and 80 kV in high-resolution TEM (HRTEM). When aligned texture was identified in TEM, samples were further subjected to HRTEM. Images were then processed using GATAN Digital Micrograph software.

## Data Availability

The data that support the findings of this study are available from the corresponding author upon reasonable request.
